# Dynamics of freshwater snails and *Schistosoma* infection prevalence in schoolchildren during the construction and operation of a multipurpose dam in central Côte d’Ivoire

**DOI:** 10.1186/s40249-017-0305-3

**Published:** 2017-05-04

**Authors:** Nana R. Diakité, Mirko S. Winkler, Jean T. Coulibaly, Négnorogo Guindo-Coulibaly, Jürg Utzinger, Eliézer K. N’Goran

**Affiliations:** 1 Laboratoire de Zoologie et Biologie Animale, Unité de Formation et de Recherche Biosciences, Université Félix Houphouët-Boigny, 22 BP 522, Abidjan 22, Côte d’Ivoire; 2 Swiss Tropical and Public Health Institute, P.O. Box, CH-4002, Basel, Switzerland; 3 University of Basel, P.O. Box, CH-4003, Basel, Switzerland; 4 Département Environnement et Santé, Centre Suisse de Recherches Scientifiques en Côte d’Ivoire, 01 BP 1303, Abidjan 01, Côte d’Ivoire

**Keywords:** Schistosomiasis, Intermediate host snail, Transmission, Water resources development and management, Multipurpose dam, Côte d’Ivoire

## Abstract

**Background:**

The construction and operation of small multipurpose dams in Africa have a history of altering the transmission of water-based diseases, including schistosomiasis. The current study was designed to investigate the abundance and dynamics of schistosomiasis intermediate host snails and *Schistosoma* infections in humans during the construction and the first years of operation of a small multipurpose dam in Côte d’Ivoire.

**Methods:**

The study was carried out in Raffierkro and four neighbouring villages in central Côte d’Ivoire between 2007 and 2012. Snails were collected by two experienced investigators using scoops and forceps for 15 min at each site. Snails were identified at genera and, whenever possible, species level, and subjected to testing for cercarial shedding. Schoolchildren aged 6–15 years were examined once every year for *Schistosoma haematobium* and *S. mansoni* infection, using urine filtration and duplication Kato-Katz thick smears, respectively. Additionally, 551 adults were examined for *Schistosoma* infection before (June 2007) and 359 individuals 2 years after dam construction (June 2009).

**Results:**

Overall, 1 700 snails belonging to nine different genera were collected from 19 sampling sites. *Bulinus* (potential intermediate host snails of *S. haematobium*) and *Pila* were the most common genera, whereas *Biomphalaria* (potential intermediate host snail of *S. mansoni*), *Lymnaea*, *Physa* and *Melanoides* were found in two villages. During the first-year sampling period, 65 snails were collected, of which 13 (20%) were schistosomiasis intermediate hosts. In subsequent years, out of 1 635 snails collected, 1 079 (66%) were identified as potential intermediate host for schistosomiasis, but none were shedding cercariae. The prevalence of *S. mansoni* among adults in the study area was low (0.4% in 2007 and 0.3% in 2009), whereas the prevalence of *S. haematobium* declined from 13.9% to 2.9% in this two-year period.

**Conclusions:**

The low prevalence of schistosomiasis in humans and the absence of infected intermediate host snails during the construction and early phase of operation of a small multipurpose dam suggest that there was no or only very little local transmission. However, the considerable increase in the number of intermediate host snails and their dispersion in irrigation canals call for rigorous surveillance, so that adequate public health measures can be taken in case of early signs of an outbreak.

**Electronic supplementary material:**

The online version of this article (doi:10.1186/s40249-017-0305-3) contains supplementary material, which is available to authorized users.

## Multilingual abstracts

Please see Additional file 1 for translations of the six official working languages of the United Nations.

## Background

In many African countries large dams have been constructed for hydropower production, while small multipurpose dams aim at enhanced agricultural production and livestock breeding [1]. Environmental transformation deriving from the construction and operation of water resources are root causes for the emergence and intensification of infectious diseases transmitted by mosquito vectors (e.g. lymphatic filariasis and malaria) and snail intermediate hosts [2–7]. In Africa, snails of the genera *Biomphalaria* and *Bulinus* act as intermediate hosts for *Schistosoma mansoni* and *S. haematobium*, respectively [8, 9]. Snail habitats are diverse and the transmission of schistosomiasis is governed by human-water contact patterns [10, 11]. Hence, communities living in close proximity to infested water bodies are at risk of schistosomiasis [12].

In Côte d’Ivoire, *Biomphalaria pfeifferi* is the sole intermediate host of *S. mansoni*, while *Bulinus truncatus* and *Bu. globosus* are transmitting *S. haematobium* [13]*.* The construction of major water resource development projects goes back to the early 1970s. For example, two large dams were constructed on the Bandama River; i.e. Lake Kossou (completed in 1972) and Lake Taabo (filled in 1979). Both dam constructions were associated with outbreaks of *S. haematobium* [14]. In addition to large dams, more than 500 small multipurpose dams were built in the northern and central parts of Côte d’Ivoire [15–17]. The aim of this study was to deepen the understanding of the presence and dynamics of freshwater snail populations in the context of a hydro-agricultural development, placing particular emphasis on intermediate host snails of schistosomiasis. For this purpose, our study covered the phases of dam construction into the first years of intensified agricultural production. The investigation was carried out in the transition zone between savannah in the north and tropical rainforest in the south of Côte d’Ivoire. Our research aims at contributing to the prediction of the transmission of schistosomiasis and informing surveillance-response systems to prevent and control schistosomiasis in areas characterised by profound environmental transformation.

## Methods

### Ethics statement

This study was approved by the institutional research commission of the Centre Suisse de Recherches Scientifiques en Côte d’Ivoire (CSRS) and received approval by Bouaké’s health authorities. In each village, permission to work was granted by local chiefs. Community members were informed in detail about the objectives, procedures, and potential risks and benefits related to the study. Illiterate people were orally informed in the local language. Written informed consent was obtained at the beginning of the study from adult participants or from parents/guardians of children aged less than 18 years old. People found with *Schistosoma* infection were treated with praziquantel (40 mg/kg of body weight) while a single oral dose of albendazole (400 mg) was given to individuals with soil-transmitted helminth infection.

### Study area and population

**Fig. 1:**
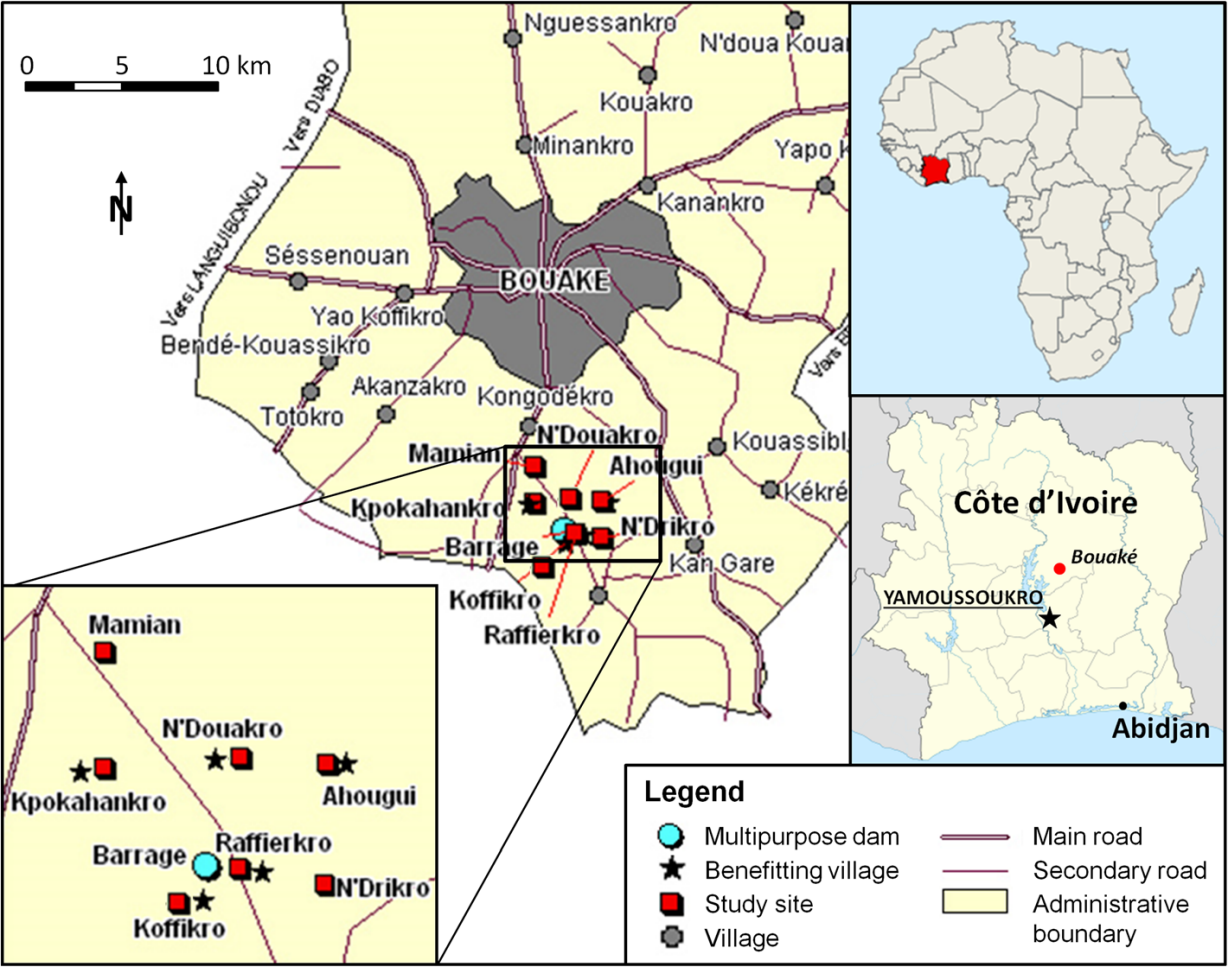
Map of study site near Bouaké, central Côte d’Ivoire

The study was carried out in five villages located in proximity to a small multipurpose dam (i.e. Ahougui, Koffikro, Kpokahankro, N’Douakro and Raffierkro) [18] and two villages without activities in proximity to the small dam (i.e. Mamian and N’Drikro). All villages were located near Bouaké, in the central part of Côte d’Ivoire (geographical coordinates 7°44’ N latitude and 5°41’ W longitude) as shown in Fig. 1.

During the study period from 2007 to 2012, the daily average temperature ranged between 23.7 °C and 33.8 °C with an average annual rainfall of 1 229 mm and 1 350 mm. The local humid tropical climate zone is characterised by two well-defined seasons: a dry season from November to February and a long rainy season from March to October. The vegetation is typical for the transition zone from tropical rainforest to savannah. Before dam construction, people were mainly engaged in subsistence agriculture (e.g. cassava and yams). Construction of the dam provided new opportunities for local dwellers, including irrigated rice cultivation, vegetable farming and inland fish production.

### Study design

**Fig. 2:**
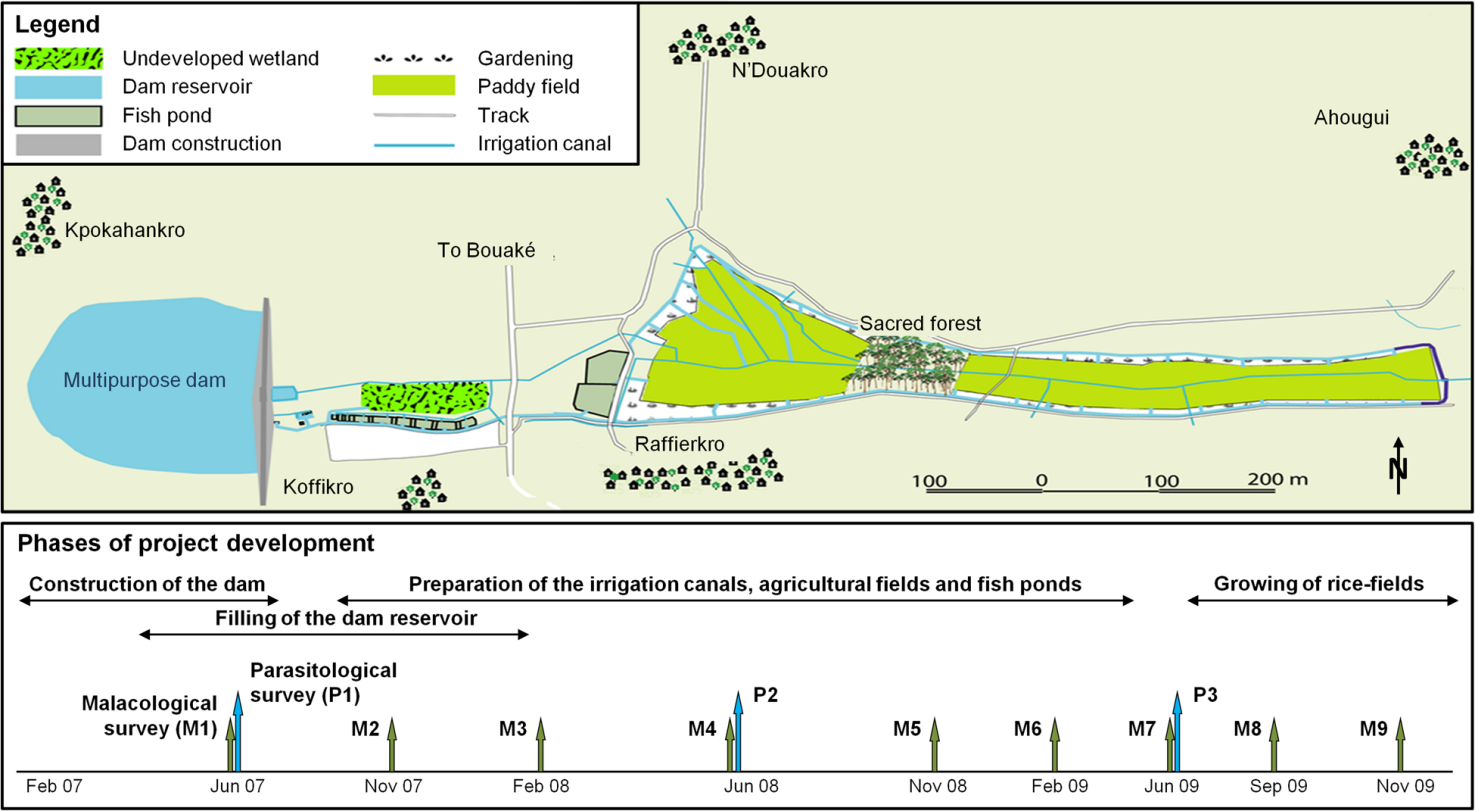
Study area near Bouaké in central Côte d'Ivoire, showing the location of the study villages within the irrigation scheme, different activities around the multipurpose dam and month of malacological and parasitological surveys

This study was designed as a repeated cross-sectional survey implemented over a six-year period. During the construction of the multipurpose dam, from June 2007 to November 2009, nine malacological (M1–M9) and three parasitological (P1–P3) surveys were carried out capturing the main phases of project development (see Fig. 2): (i) the construction of the dam; (ii) the construction of the irrigation canals, agricultural fields and fish ponds; and (iii) the growing of rice-fields. In the early stages of the dam operation, the parasitological surveys were continued from 2010 to 2012 (P4–P6; in the months of June/July) for the follow up.

### Malacological surveys

In each study village, all the water contact sites where people used to collect water, wash clothes, bath, swim and play (young children) were surveyed. Snail sampling was undertaken by two experienced field collectors using scoops and forceps for the duration of 15 min. Snail habitats were characterised using a standard form. The collected snail specimens were transferred in perforated plastic boxes to a nearby laboratory. Snails were identified according to shell morphology and structure using standard identification keys [19, 20]. After genus and/or species identification, potential intermediate host snails were tested over a one-month period for *Schistosoma* cercaria shedding in their swimming water after two–three hours of exposure to sunlight during five times points: the day of collection and 1, 2, 3 and 4 weeks after collection. Subsequently cercariae shedded were morphologically identified through identification keys put forward by Frandsen and Christensen [21].

### Parasitological survey

A total sample size of 600 individuals was the set target for the parasitological survey, representing 50% of the total population in the study area. For accounting for the different population numbers in the study villages, sampling proportional to size was applied. Households were randomly selected from previously prepared household list and children aged 6–15 years attending primary school were invited to participate in the survey. In addition, all primary schoolchildren from Raffierkro, N’Drikro and Mamian were screened. After informed consent, stool and urine samples were collected with the assistance of local nurses. Two plastic containers (125 ml) were distributed to each adult participant the day before the sample collection. Participants were invited to submit a sample of their own morning stool and urine in two separate collection containers. Each participant was given a unique identification number. The samples obtained were then transferred to a nearby laboratory and worked**-**up the same day for *S. haematobium* (urine), *S. mansoni* and soil-transmitted helminths (stool) using standardised, quality-controlled methods. In brief, stool samples were processed by the Kato-Katz technique, producing two thick smears per stool per participant [22]. After a clearing time of 30–45 min, Kato-Katz thick smears were examined under a microscope by experienced laboratory technicians. Helminth eggs were counted and recorded on a species-specific level. Individual egg output was expressed as eggs per gram of faeces (EPG), which was calculated as the arithmetic mean egg count from two readings multiplied by a factor of 24. The prevalence and intensity of *S. haematobium* infection were estimated based on a urine filtration method. In brief, 10 ml of urine was pressed through a polyamide (Nytrel) filter (mesh size: 20 μm) and stained with a drop of Lugol’s solution for subsequent microscopic examination. The intensity of *S. haematobium* infection was expressed as the number of eggs per 10 ml of urine.

### Statistical analysis

Double entry of data using Excel (Microsoft; Redmond, WA, USA) was done and cross-checked with EpiInfo version 3.2 (Centers for Disease Control and Prevention; Atlanta, GA, USA). The final database was analysed using STATA version 9.0 (Stata Corporation, College Station, TX, USA). The Kruskal-Wallis (KW) test was employed to compare the differences in snail densities among villages. Pearson’s *χ* ^2^ test was used to compare proportions between villages, age categories and sex. The Student’s *t-*test was used to compare means. For all statistics, a *P* value below 0.05 was considered as statistically significant.

## Results

### Water contact sites and snail composition

A total of 20 human water contact sites were recorded: eight in Kpokahankro, five in Raffierkro, three in Ahougui and N’Douakro each and one in Koffikro. A total of 1 700 freshwater snails were collected from the sampling sites (Table 1). Identification based on shell morphology revealed nine genera. Snails belonging to the genera *Bulinus* and *Pila* were observed in all the localities, whereas the genera *Biomphalaria*, *Lymnaea*, *Physa* and *Melanoides* were observed in only two localities (Ahougui and Raffierkro). In the early construction phase (year 2007), 65 snails belonging to three species were collected. *Bi. pfeifferi* in Ahougui, *Bu. forskalii* in Kpokahankro and Raffiekro and *P. acuta* in Raffiekro. During this period the intermediate host *Bi. pfeifferi* was observed only in Ahougui with 13 individuals.

**Table 1:** Snail abundance in a study carried out near Bouaké in central Côte d'Ivoire between June 2007 and November 2009, stratified by study period and village

	Ahougui			Kpokahankro			N’Douakro			Raffierkro			Total
Species	P1	P2	P3	P1	P2	P3	P1	P2	P3	P1	P2	P3	
*Biomphalaria pfeifferi*	13	0	0	0	0	0	0	0	0	0	660	52	725
*Bulinus truncatus*	0	0	3	0	0	0	0	0	0	0	105	54	162
*Bu*linus *globosus*	0	0	0	0	0	0	0	0	0	0	116	89	205
*Bulinus forskalii*	0	11	21	12	55	50	0	0	5	15	35	10	214
*Lymnaea natalensis*	0	0	3	0	0	0	0	0	0	0	7	5	15
*Physa acuta*	0	0	3	0	0	0	0	0	0	25	30	3	61
*Planorbe sp*	0	20	1	0	7	13	0	0	0	0	0	0	41
*Pila africana*	0	10	24	0	9	6	0	0	1	0	51	0	101
*Segmenorbis* sp	0	0	0	0	0	0	0	0	3	0	0	0	3
*Melanoides tuberculata*	0	120	16	0	0	0	0	0	0	0	23	13	172
*Lanistes* sp	0	1	0	0	0	0	0	0	0	0	0	0	1
Total	13	162	71	12	71	69	0	0	9	40	1 027	226	1 700

Koffikro: no water contacts points

In 2008 (advanced construction phase), 1 260 snails, including the three intermediate hosts *Bi. pfeifferi*, *Bu. truncatus* and *Bu. globosus*, were collected. Most of them (*n* = 1 027; 81.5%) were observed in Raffierkro. In the same village *Bi. pfeifferi* was the predominant species (64.3%), followed by *Bu. globosus* (11.3%) and *Bu. truncatus* (10.2%). In 2009 (late construction phase), the same species were observed, though with a decrease in the number of individuals for each of the three species.

Over the entire malacological study period (June 2007–November 2009), the highest number of intermediate host snails was recorded in Raffierkro. *Bi. pfeifferi* was the most frequently encountered intermediate host snail in Raffierkro (*n* = 725; 42.6%), followed by *Bu. globosus* (*n* = 205; 12.1%) and *Bu. truncatus* (*n* = 214; 12.6%). *M. tuberculata* was the predominant snail species in Ahougui (*n* = 136; 55.3%), while *Bu. forskalii* dominated in Kpokahankro (*n* = 117; 77.0%) and N’Douakro (*n* = 5; 55.3%). In Ahougui, few *Bi. pfeifferi* (*n* = 13) and *Bu. truncatus* (*n* = 3) were collected during the first and the third year of the study, respectively.

### Habitats and density of schistosomiasis intermediate host snails

The key habitats where snails were observed consisted of canals, the dam, paddies, swamps and water reservoirs. The mean snail abundance varied significantly across the four villages (KW = 136.5; *P* = 0.025), with Raffierkro recording the highest abundance.

**Fig. 3:**
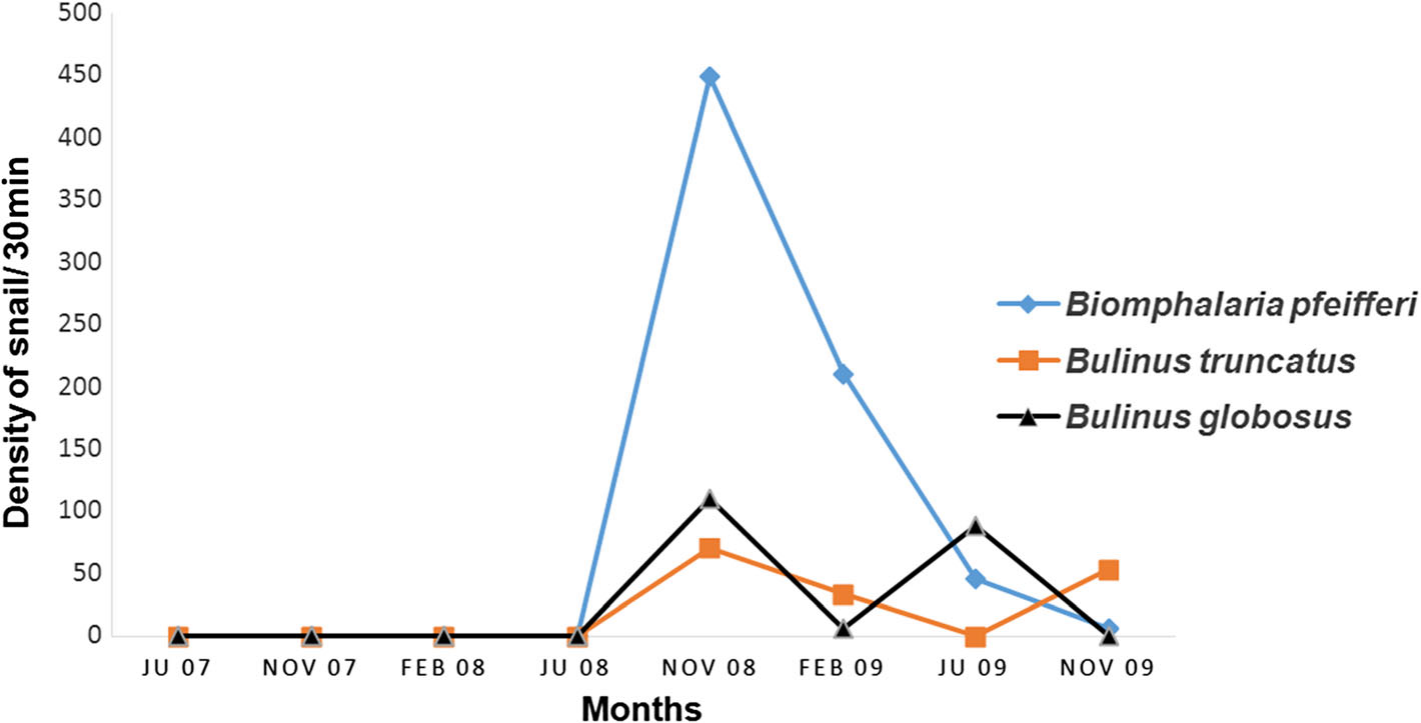
Density of the three intermediates host snail species of schistosomiasis in Raffierkro from June 2007 to November 2009

The density of schistosomiasis intermediate host snails was highest in Raffierkro. As shown in Fig. 3, during the second time-period, from June to November 2008, the number of *Bi. pfeifferi* increased rapidly from 0 to 449 snails. Subsequently, numbers decreased progressively from 211 to 46 and six snails from February to November 2009. With regard to *Bu. globosus*, two peaks of relative abundance were observed in November 2008 and June 2009, while *Bu. truncatus* showed only one peak (November 2008). No *Bu. globosus* snails were observed in February 2009, while *Bu. truncatus* population decreased. The maximum abundances of *Bu. globosus* (*n* = 89) and *Bu. truncatus* (*n* = 54) individuals were observed, in June and November 2009, respectively. Overall, density of snails collected in Ahougui was lower compared to Raffierkro.

### Infection of snails

None of the snails was found shedding schistosome cercariae. Yet, some snails were found shedding zoophilic cercariae, such as Strigeides, and Xiphidio-cercariae.

### Study adherence and population characteristics

In June 2007, stool and urine samples could be obtained from 534 and 551 people, respectively. In the last survey round conducted in June 2009, 389 and 359 stool and urine samples were obtained, respectively. The study population was mainly composed of individuals above 15 years (54.8%). From June 2007 to June 2012, all primary schoolchildren from Raffierkro, N’Drikro and Mamian were screened.

### Prevalence and intensity of *Schistosoma* infections by sex and age

The cumulative prevalence for the two *Schistosoma* species in the five study villages was significantly lower in 2009 (2.6%, 95% *CI*: 1.1–5.0%), compared to the baseline cross-sectional survey in 2007 (7.5%, 95% *CI*: 5.4–10.1%). This difference was statistically significant (*χ* ^2^ = 7.97; *P* = 0.005). In Table 2, the overall prevalence of *Schistosoma* infection in individual villages was below 6.0% at the time of the baseline assessment in 2007. Prevalences remained at similar levels during the two follow-up surveys, except for Raffierkro, where a higher prevalence was observed in 2007 (*P* = 0.005), associated with a difference in the prevalence between males (10.5%) and females (4.9%) (*χ* ^2^ = 5.88; *P* = 0.024). In the dam area, the global prevalence observed of *S. mansoni* was very low at 0.4% in 2007 and 0.3% in 2009. No infected people were observed in Ahougui, Koffikro, N’Douakro and Kpokahankro. In Raffierkro, the prevalence decreased from 1.3% to 0.7% from 2007 to 2009, respectively. All cases detected were restricted to school-aged children (6–15 years) (Table 3). The intensity of infection was very low in both 2007 and 2009 (0.01 EPG, 95% *CI*: 0–0.03 EPG).

**Table 2:** Prevalence of *S. haematobium* and *S. mansoni* in the study villages near Bouaké in central Côte d'Ivoire in proximity to dam between June 2007 and November 2009

	2007				2009			
	*S. haematobium*		*S. mansoni*		*S. haematobium*		*S. mansoni*	
Village	*n*	p (%)	*n*	p (%)	*n*	p (%)	*n*	p (%)
Ahougui	101	3 (2.9)	99	0	83	1 (1.2)	85	0
Koffikro	91	3 (3.3)	90	0	45	2 (4.4)	51	0
Kpokahankro	110	4 (3.6)	109	0	65	1 (1.5)	73	0
N’Douakro	87	3 (3.5)	79	0	35	0	38	0
Raffierkro	162	25 (15.4)	157	2 (1.3)	131	4 (3.1)	142	1 (0.7)
Total	551	38 (6.9)	534	2 (0.4)	359	8 (2.2)	389	1 (0.3)

*n*: number of individuals examined

p: number of individuals infected

%: prevalence

**Table 3:** Prevalence of *S. haematobium* in the five study villages near Bouaké in central Côte d'Ivoire, stratified by age category between 2007 and 2009

	2007		2009			
Age category (in years)	*n*	*p* (%)	*n*	*p* (%)	*χ* ^2^	*P-*value
≤5	38	3 (7.9)	36	1 (2.7)		
6–15	198	23 (11.6)	140	4 (3.0)		
>15	315	12 (3.8)	183	3 (1.6)		
Total	551	38 (6.9)	372	8 (2.2)	7.40	0.006

*n*: number of individuals examined

p: number of individuals infected

%: prevalence

The prevalence of *S. haematobium* was also low, decreasing from 6.9% (95% *CI*: 4.7–9.0%) in 2007 to 2.2% (95% *CI*: 0.7–3.8%) in 2009 (*χ* ^2^ = 9.86; *P* = 0.002). Although the prevalence was low, a significant sex difference was observed. In 2007, males showed a significantly higher prevalence than female (9.6% *versus* 4.7%; *χ* ^2^ = 5.20; *P* = 0.022). In 2009, the prevalence remained comparable (*χ* ^2^ = 0.38; *P* = 0.54), except for Raffierkro, where a prevalence of 15.4 and 3.1% was recorded in 2007 and 2009, respectively

The geometric mean of excreted eggs/10 ml of urine was low throughout, with an observed decrease from 0.15 eggs/10 ml (95% *CI*: 0.09–0.21 eggs/10 ml) in 2007 to 0.05 eggs/10 ml (95% *CI*: 0.01–0.10 eggs/ml) in 2009 (*P* = 0.70).

Considering schoolchildren in 2007, a difference was observed in the prevalence of *S. haematobium* between Raffierkro and the two schools of Mamian and N’Drikro. The prevalence of infection in Raffierkro’s school decreased from 13.9% (95% *CI*: 8.3–19.4%) to 2.9% (95% *CI*: 0.4–6.2%) in 2009. During the 2010 and 2011 cross-sectional surveys, no *Schistosoma* infection was observed in the three schools. In 2012, only one and four cases were observed in Raffierkro and N’Drikro, respectively (Table 4). All of these cases were detected in children aged between six and ten years.

**Table 4:** Prevalence of schistosomiasis in children 6–15 years in the five study villages near Bouaké in central Côte d'Ivoire from 2007 to 2012

			Sex				
			Female		Male		
School	*n*	*p* (%)	*n*	*p* (%)	*n*	*p* (%)	*P*-value
2007 (P1: 24–30 June)							
Raffierkro	151	21 (13.9)	56	7 (12.5)	95	14 (14.7)	0.701
Mamian	22	0	0	0	0	0	-
N’Drikro	28	0	0	0	0	0	-
2008 (P2: 28 May–3 June)							
Raffierkro	184	8 (4.3)	81	2 (2.4)	103	6 (5.8)	0.268
Mamian	77	1 (1.3)	28	0	49	2	0.447
N’Drikro	100	4 (4.0)	50	6	50	2	0.327
2009 (P3: 21–25 June)							
Raffierkro	138	4 (2.9)	61	2 (3.3)	77	2 (3.8)	0.852
Mamian	113	0	0	0	0	0	-
N’Drikro	115	2 (1.8)	61	1 (1.6)	54	1 (1.9)	0.932
2010 (P4: 27 May–1 June)							
Raffierkro	154	0	60	0	94	0	-
Mamian	103	0	43	0	60	0	-
N’Drikro	164	0	70	0	94	0	-
2011 (P5: 10–15 July)							
Raffierkro	239	0	96	0	143	0	-
Mamian	94	0	40	0	54	0	-
N’Drikro	159	0	74	0	85	0	-
2012 (P6: 30 May–3 June)							
Raffierkro	155	3 (1.9)	60	1 (1.6)	95	2 (2.1)	
Mamian	72	0	28	0	43	0	
N’Drikro	86	5 (5.8)	44	9.09	42	2.38	

*n*: number of individuals examined

p: number of individuals infected

%: prevalence

## Discussion

### Abundance of snails

The current study aimed to deepen our understanding of the variation of freshwater snail populations in central Côte d’Ivoire in the face of the construction and operation of a small, multipurpose dam. We observed a considerable increase in the abundance of snails during the second observation period, i.e. after the dam had been filled. The diversity of freshwater snails and the exponential growth of *Bi. pfeifferi* are probably explained by a more stable hydrological system [23]. All the snails collected belonged to the same genera and species that have been reported before in the greater Bouaké area [24] with the exception of *Physa acuta* that had not been reported before. *Bu. truncatus* was present at the level of the dam reservoir but absent in the other villages having water supply points and being drained completely during the dry season. *Bu. truncatus* is a cosmopolitan species with a preference for sunlight, clean, stagnant and permanent aquatic environments [19]. The construction of a multipurpose dam and formation of a small man-made lake support the proliferation of *Bu. truncatus*. In a preceding study by Poda in Burkina Faso it was noted that dams account for 61.7% of the biotopes with *Bu. truncatus* and seem the preferential habitats of this species [8]. This observation is similar to that described in our context. In contrast, *Bu. globosus* prefers cluttered areas and low water flow such as in an irrigated area [25, 26].

### Cercarial shedding and schistosomiasis transmission

In spite of very high abundance of snails and prolonged exposure (up to 1 month) to check for cercarial shedding, none of the snails collected shed cercariae and the schistosome infection level among school children and adults was low. Indeed, with a *Schistosoma* infection prevalence below 10%, all the localities surveyed are considered hypoendemic [27]. This observation suggests that the high abundance of intermediate host snails does not correlate with human infection. These results are consistent with other studies from endemic areas in Uganda and Kenya; showing a high abundance of snails but no or only very few snails shedding cercariae [28, 29]. Indeed, the quasi-absence of cercariae shedding might explain the low human prevalence. The absence of infection might be due to health education received by parents and children, who emphasised avoidance of water contact such as bathing, washing clothes or kitchen utensils in the newly formed water resource. The surveys undertaken confirmed the low prevalence observed. Hence, it is conceivable that there was no local transmission of schistosomiasis at the time of the study despite suitable snail habitats. The few infected people might have acquired their infections elsewhere.

*S. mansoni* was rarely found in the study area. This result is consistent with observations made before in villages in close proximity to large dams, namely Lake Kossou and Lake Taabo. However, within a few years of construction of these two large dams, there were outbreaks of *S. haematobium*, prevalence increased from 14% to 53% and from 0% to 73% in the two dams, while *S. mansoni* was absent or present at very low rate [14]. Our study confirms that Central region of Côte d’Ivoire continues to have active transmission of *S. haematobium*. The fall of the prevalence of schistosomiasis in all the study localities might be associated with the absence of indigenous transmission and the treatment of diagnosed cases. Of note, all positive individuals were treated with praziquantel (40 mg/kg), which, according to WHO, results in cure rates ranging between 60% and 90% for *Schistosoma* infection [30, 31].

Our study illustrates the complex interactions between dam constructions, snail ecology and schistosomiasis transmission. Therefore, before any dam construction and throughout the life course of such water resources development projects, malacological and epidemiological survey must be implemented to continuously evaluate potential risks. Integrated control measures, including information, education and communication (IEC), environment management and access to treatment must be considered. Control and preventive measures that are readily tailored to specific settings are necessary to keep human infections and morbidity low.

## Conclusions

The construction and operation of a small, multipurpose dam in Raffierkro in central Côte d’Ivoire is the likely cause of a high increase of intermediate host snail fauna. *Bi. pfeifferi* was the prominent intermediate host of schistosomiasis, followed by *Bu. truncatus* and *Bu. globosus*. In spite of the presence of dam and the high abundance of intermediate host snails, no evidence of local transmission of schistosomiasis was observed. Hence, adequate control and preventive measures in this context by IEC, and rigorous surveillance is necessary, so that early signs of emergences can be addressed by public health measures.

## Additional file

Additional file 1: Multilingual abstracts in the six official working languages of the United Nations. (PDF 786 kb)

## Abbreviations

CI: Confidence interval
CSRS: Centre Suisse de Recherches Scientifiques en Côte d’Ivoire
DBL: Danish Bilharziasis Laboratory
FAO: Food and Agriculture Organization
KW: Kruskal-Wallis
WHO: World Health Organization
