# Supplementary material for: Dynamics of freshwater snails and Schistosoma infection prevalence in schoolchildren during the construction and operation of a multipurpose dam in central Côte d’Ivoire
Source: Infect Dis Poverty. 2017 May 4;6:93. doi: 10.1186/s40249-017-0305-3 (PMC5415719; doi:10.1186/s40249-017-0305-3)
Supplement: Additional file 1: — Multilingual abstracts in the six official working languages of the United Nations. (PDF 786 kb) [file 40249_2017_305_MOESM1_ESM.pdf]

Translation of the abstract into the five official working languages of the United Nations

ديناميات القواقع في المياه العذبة وانتشار عدوى البلهارسيا في المدارس خلال بناء وتشغيل سد متعدد الأغراض في وسط كوت ديفوار

نانا ر دياكيتي، ميركو س. وينكلر، جان ت. كوليبالي، نيجنوروجوجويندو-كوليبالي، يورج أوتزنجر، إلييزر ك. نجوران

الملخص

خلفية: بناء وتسبب تشغيل السدود متعددة الأغراض الصغيرة في أفريقيا منذ إنشائها في تغيير انتقال الأمراض القائمة انتقالها على المياه، بما في ذلك مرض البلهارسيا. وقد تم وضع هذه الدراسة لدراسة وفرة وديناميكية قواقع البلهارسيا الوسيطة المضيفة وإصابات البلهارسيا في البشر خلال البناء والسنوات الأولى من تشغيل سد صغير متعدد الأغراض في كوت ديفوار.

الأساليب: وقد أجريت الدراسة في رافيركرو وأربع قرى مجاورة في وسط كوت ديفوار بين عامي 2007 و 2012. تم جمع القواقع من قبل اثنين من المحققين ذوي الخبرة باستخدام المجارف وملقط لمدة 15 دقيقة في كل موقع. وقد تم تحديد القواقع وفقا للجنس، وكلما كان ذلك ممكنا، مستوى النوع، وأخضعت لاختبار إسقاط المذنبات. تم فحص تلاميذ المدارس الذين تتراوح أعمارهم بين 6 و15 عاما مرة واحدة كل عام لبيان إصابتهم بالبلهارسيا البولية و البلهارسيا المعوية من عدمه، باستخدام ترشيح البول وأسلوب كاتو-كاتز، على التوالي. بالإضافة إلى ذلك، تم فحص 551 من البالغين لعدوى البلهارسيا قبل (يونيو 2007) و 359 أفراد بعد سنتين من بناء السد (يونيو 2009).

النتائج: عموما، تم جمع 1700 قوقعة تنتمي إلى تسعة أجناس مختلفة من 19 موقع أخذت منه العينات. بولينس (القواقع التي تقوم بدور العائل الوسيط المحتمل لل بلهارسيا البولية) و Pila كانت الجنس الأكثر شيوعا، في حين بيومفلاريا (الحلزون العائل المحتمل الوسيط ل لبلهارسيا المعوية)، Physa، Lymnaea و Melanoides تم العثور عليها في قريتين. خلال فترة أخذ العينات في السنة الأولى، تم جمع 65 قوقعة، منها 13 (20.0٪) من العائل الوسيط للبلهارسيا. في السنوات اللاحقة، من أصل 1635 قوقعة تم جمعها، 1079 (66.0٪) تم تحديدها باعتبارها عائل وسيط محتمل لمرض البلهارسيا، ولكنها لم تكن تسقط السركاريا. انتشار البلهارسيا المعوية بين البالغين في منطقة الدراسة كان منخفضا (0.4٪ في عام 2007 و 0.3٪ في عام 2009)، في حين أن معدل انتشار البلهارسيا البولية انخفض من 13.9٪ إلى 2.9٪ في هذه الفترة البالغة سنتين.

الاستنتاجات: يشير انخفاض معدل انتشار مرض البلهارسيا في البشر وعدم وجود العائل الوسيط المتمثل في القواقع المصابة خلال مرحلة الإنشاء والمرحلة الأولى من تشغيل السد الصغيرمتعدد الأغراض إلى عدم وجود انتقال أو وجود انتقال ضئيل محلي فقط. ومع ذلك، فإن الزيادة الكبيرة في عدد القواقع المضيفة الوسيطة وتوزعها في قنوات الري تدعو إلى مراقبة صارمة، بحيث يمكن اتخاذ تدابير الصحة العامة الملائمة في حالة ظهور علامات مبكرة للفاشية.

Translated from English version into Arabic by Mahmoud Sami, through

TRANSLATORS WITHOUT BORDERS

在科特迪瓦中部一个多功能大坝的修建和运行期间淡水钉螺和学龄儿童血吸虫感染率的动态

Nana R. Diakité, Mirko S. Winkler, Jean T. Coulibaly, Négnorogo Guindo-Coulibaly, Jürg Utzinger, Eliézer K. N’Goran

摘要

引言：非洲小型多用途水坝的建设和运营改变了包括血吸虫病在内的水源性疾病的传播。本

研究旨在调查科特迪瓦一个小型多用途水坝建设和第一年运行期间，血吸虫的中间宿主钉螺丰度和人群感染血吸虫的动态。
方法：2007-2012 年，本研究在科特迪瓦中部 Raffierkro 和邻近的 4 个村庄进行。由两名经验丰富的研究人员在每个站点使用收集器和镊子进行 15 min 的钉螺收集。对钉螺鉴定至属，如有可能，鉴定至种，并进行逸蚴实验。6-15 岁学龄儿童每年进行一次的埃及血吸虫和曼氏血吸虫感染筛查，分别采用尿液过滤和重复的加藤厚涂片非法。此外，在 2007 年 6 月大坝建成之前对 551 名成年人，和大坝建成 2 年后（2009 年 6 月）对 359 人进行血吸虫感染检查。
结果：总体而言，从 19 个采样点收集的 1700 个钉螺属于 9 个属。小泡螺属（*Bulinus*，潜在的埃及血吸虫中间宿主钉螺）和瓶螺属（*Pila*）是最常见的属，而在两个村庄发现了双脐螺属（*Biomphalaria*，潜在的曼氏血吸虫中间宿主钉螺）、椎实螺属（*Lymnaea*）、膀胱螺属（*Physa*）和拟黑螺属（*Melanoides*）。在第一年的采样期间，收集到 65 只钉螺，其中 13 只（20%）为血吸虫病中间宿主。在随后的几年中，在收集的 1635 只钉螺中，其中 1079 只（66%）被确定为潜在的血吸虫病中间宿主，但未发现逸出的尾蚴。在所研究地区成年人的曼氏血吸虫病患病率低（2007 年为 0.4%，2009 年为 0.3%），而在这两年期间埃及血吸虫的患病率从 13.9%下降到 2.9%。
结论：在一个小型多功能大坝的建设和早期运营期间，人群血吸虫病患病率低以及没有感染性钉螺，表明没有或者很少有血吸虫病的本地传播。然而，需要严格监测中间宿主钉螺在数量上的增加以及它们在灌溉渠的分布，以便在发生早期迹象的情况下采取适当的公共卫生措施。

Translated from English version into Chinese by Yin-Long Li, edited by Pin Yang

CHINESE CENTER FOR DISEASE CONTROL AND PREVENTION
NATIONAL INSTITUTE OF PARASITIC DISEASES

Dynamique des escargots d'eau douce et prévalence de la *schistosomiase* chez les écoliers pendant la construction et l'exploitation d'un barrage polyvalent dans le centre de la Côte d'Ivoire

Nana R. Diakité, Mirko S. Winkler, Jean T. Coulibaly, Négnorogo Guindo-Coulibaly, Jürg Utzinger, Eliézer K. N'Goran

Résumé
Contexte: la construction et l'exploitation de petits barrages polyvalents en Afrique ont tendance à altérer la transmission des maladies hydriques, dont la schistosomiase. La présente étude vise à évaluer l'abondance et la dynamique des escargots, hôtes intermédiaires de la schistosomiase, et l'infection du *Schistosoma* chez l'homme lors de la construction et au cours des premières années d'exploitation d'un petit barrage polyvalent en Côte d'Ivoire.
Méthodes: L'étude a été réalisée à Raffierkro et dans quatre villages voisins dans le centre de la Côte d'Ivoire entre 2007 et 2012. Les escargots ont été collectés par deux enquêteurs expérimentés à l'aide de petites pelles et de pinces, pendant 15 minutes sur chaque site. Les escargots ont été identifiés par genre et, lorsque possible, par espèce, puis ont été soumis à des tests d'émission de cercaires. Des écoliers âgés de 6 à 15 ans ont été examinés une fois par an pour *l'infection*

Schistosoma haematobium et *S. mansoni* par filtration d'urine et par duplication de la technique Kato-Katz (étalement épais de selles sous cellophane), respectivement. De plus, 551 adultes ont préalablement été examinés pour l'infection *Schistosoma* (en juin 2007). 359 personnes ont également été examinées deux ans après la construction du barrage (juin 2009).
Résultats: Au total, 1700 escargots appartenant à neuf genres différents ont été collectés sur les 19 sites de prélèvement. *Bulinus* (des escargots hôtes intermédiaires potentiels du*S. haematobium*) et *Pila* sont les genres plus communs, tandis que *Biomphalaria* (escargots hôtes intermédiaires potentiels du *S. mansoni*), *Lymnaea*,*Physa* et*Melanoides*ont été trouvés dans deux villages. Au cours de la période d'échantillonnage de la première année, 65 escargots ont été capturés, dont 13 (20,0 %) étaient les hôtes intermédiaires de schistosomiase. Dans les années suivantes, sur 1 635 escargots collectés, 1.079 (66%) ont été identifiés comme hôte intermédiaire potentiel pour la schistosomiase, mais aucun n'a émis de cercaire. La prévalence de la *S. mansoni* chez les adultes dans la zone d'étude est faible (0,4% en 2007 et 0,3% en 2009), tandis que la prévalence de la *S. haematobium* a diminué, chutant de 13,9% à 2,9% pendant cette période de 2 ans.
Conclusions: La faible prévalence de la schistosomiase chez l'humain et l'absence d'escargot en tant que hôte intermédiaire infecté pendant la construction et la phase préliminaire d'exploitation du barrage polyvalent suggère qu'il n'y a pas, ou très peu de risque de transmission locale. Toutefois, l'augmentation considérable du nombre d'escargots hôtes intermédiaires et leur dispersion dans les canaux d'irrigation appellent à une surveillance accrue, afin que les mesures de santé publique adéquates puissent être prises dès les premiers signes d'une épidémie.

Translated from English version into French by Laurence Masing, through

TRANSLATORS
WITHOUT BORDERS

Динамика распространения пресноводных улиток и инфекции *Schistosoma* у детей школьного возраста в период строительства и эксплуатации многофункциональной дамбы в центральной части Кот-д'Ивуара.

Нана Р. Диаките, Мирко С. Уинклер, Жан Т. Кулибали, Негнорого Гуиндо-Кулибали, Юрг Уцингер, Элиэзер К. Н'Горан

Краткое изложение
Вводная информация. Строительство и эксплуатация малых многофункциональных дамб в Африке уходит своими корнями в изменение распространения заболеваний, передающихся с помощью воды, включая шистосомоз. Данное исследование было разработано с целью изучения численности и динамики распространения улиток, как промежуточного хозяина инфекций, вызванных *Schistosoma,* в том числе у людей, в ходе строительства и первых лет эксплуатации небольшой многофункциональной дамбы в Кот-д'Ивуаре.
Методика. Исследование проводилось в деревне Раффиеркро и четырех соседних деревнях в центральной части Кот-д'Ивуара в период с 2007 по 2012 гг. Сбором улиток занимались два

опытных исследователя, которые действовали с помощью совков и пинцета в течение 15 минут на каждом участке. Улитки идентифицировались по родам и, по возможности, видам и проверялись на выделение церкарий. Раз в год школьников в возрасте от 6 до 15 лет проверяли на наличие инфекций *Schistosoma haematobium* и *S. mansoni* с помощью фильтрации мочи и метода толстого мазка по Като соответственно. Кроме того, на наличие инфекции *Schistosoma* было обследовано 551 взрослый человек до (июнь 2007 г.) и 359 человека через 2 года после строительства дамбы (июнь 2009 г.).

Результаты. В общей сложности, на 19 площадках по отбору образцов было собрано 1700 улиток, принадлежащих к 9 разным родам. *Bulinus* (улитка, являющаяся потенциальным промежуточным хозяином *S. haematobium*) и *Pila* оказались самыми многочисленными, в то время как *Biomphalaria* (улитка, являющаяся потенциальным промежуточным хозяином *S. mansoni*), *Lymnaea*, *Physa* и *Melanoides* были обнаружены в двух деревнях. В течение первого года периода выборки было собрано 65 улиток, из которых 13 (20,0%) были промежуточными хозяевами инфекции шистосомоз. В последующие годы из 1635 собранных улиток 1079 (66,0%) особей были определены в качестве потенциального промежуточного хозяина инфекции шистосомоз, но никто из них не выделял церкарий. Распространение *S. mansoni* у участвующих в исследовании взрослых было низким (0,4% 2007 г. и 0,3% в 2009 г.), в то время как распространение *S. haematobium* за те же 2 года упало с 13,9% до 2,9%.

Выводы. Низкое распространение шистосомоза у людей и отсутствие зараженных промежуточных хозяев — улиток на этапе строительства и первого этапа эксплуатации небольшой многофункциональной дамбы говорят о том, что местная передача отсутствовала или находилась на очень низком уровне. Тем не менее, значительное увеличение числа промежуточных улиток-хозяев и их дисперсия в оросительных каналах требуют строгого надзора, что позволит обеспечить реализацию адекватных мер медицинского контроля в случае первых признаков вспышки.

Translated from English version into Russian by Irina Zayonchkovskaya, through

TRANSLATORS WITHOUT BORDERS

Dinámica de los caracoles de agua dulce y el predominio de infección por Esquistosoma en niños en edad escolar durante la construcción y funcionamiento de una presa con múltiples propósitos en la parte del centro de Costa de Ivoria

Nana R. Diakité, Mirko S., Winkler, Jean T. Coulibaly, Négnorogo Guindo-Coulibary, Jürg Utzinger, Eliézer K. N'Goran

Resumen

Fondo: La construcción y funcionamiento de pequeñas presas para usos múltiples en África tiene un historial de alterar la transmisión de enfermedades por agua, incluyendo la esquistosomiasis. El presente estudio fue diseñado para investigar la abundancia y la dinámica de la esquistosomiasis por

intermediación de los huéspedes y las infecciones por Esquistosoma en los seres humanos durante la construcción y los primeros años de funcionamiento de una pequeña presa para usos múltiples en Costa de Marfil.

métodos: El estudio se llevó a cabo en Raffierkro y cuatro pueblos vecinos en el centro de Costa de Marfil entre 2007 y 2012. Dos investigadores experimentados recogieron caracoles usando palas y pinzas durante 15 minutos en cada sitio. Los caracoles fueron identificados por géneros y, siempre que fue posible, por especie, y se sometieron a pruebas por derramamiento de cercarias. Los escolares de 6 - 15 años fueron examinados una vez al año para encontrar infección por Schistosoma haematobium y S. mansoni, mediante la filtración de orina y la duplicación gotas gruesas Kato-Katz, respectivamente. Además, se examinaron 551 adultos para infección por Schistosoma anteriormente (junio de 2007) y 359 individuos 2 años después de la construcción de presas (junio de 2009).

resultados: En general, se recogieron 1.700 caracoles pertenecientes a nueve géneros diferentes de 19 sitios de muestreo. Bulinus (potenciales caracoles huéspedes intermedios de S. haematobium) y Pila fueron los géneros más comunes, mientras que en dos aldeas se encontraron biomphalaria (Caracol huésped intermediario potencial de S. mansoni), Lymnaea, Physa y Melanoides. Durante el primer año del período de muestreo, se recogieron 65 caracoles, de los cuales 13 (20,0%) eran huéspedes intermiediarios de esquistosomiasis. En los años siguientes, de cada 1.635 caracoles recogidos, 1.079 (66,0%) fueron identificados como huéspedes intermediario potenciales de esquistosomiasis, pero ninguno fue derramando cercarias. El predominio de S. mansoni entre los adultos en el área de estudio fue baja (0,4% en 2007 y 0,3% en 2009), mientras que el predominio de S. haematobium disminuyó de 13,9% a 2,9% en ese período de 2 años.

conclusiones: El bajo predominio de esquistosomiasis en los seres humanos y la ausencia de caracoles huéspedes intermedios infectados durante la construcción y la fase inicial de funcionamiento de una pequeña presa para usos múltiples sugiere que no había ninguna o muy poca transmisión local. Sin embargo, el aumento considerable del número de caracoles huéspedes intermedios, y su dispersión en los canales de riego hace un llamamiento a la necesidad de supervisión rigurosa, por lo que pueden tomarse medidas de salud pública adecuadas en el caso de los primeros signos de un brote.

Translated from English version into Spanish by ISABEL SANLLEHI, through

TRANSLATORS
WITHOUT BORDERS
